# Creating superhydrophobic and antibacterial surfaces on gold by femtosecond laser pulses

**DOI:** 10.1016/j.apsusc.2019.144952

**Published:** 2020-03-15

**Authors:** Sohail A. Jalil, Mahreen Akram, Javeed A. Bhat, Jeffrey J. Hayes, Subhash C. Singh, Mohamed ElKabbash, Chunlei Guo

**Affiliations:** a The Institute of Optics, University of Rochester, Rochester, NY 14627, USA; b GPL State Key Laboratory of Applied Optics, Changchun Institute of Optics, Fine Mechanics and Physics, Chinese Academy of Sciences, Changchun 130033, China; c University of Chinese Academy of Sciences, Beijing 100049, China; d Centre for Advanced Studies in Physics, Government College University, Lahore 54000, Pakistan; e Department of Biochemistry and Biophysics, University of Rochester Medical Center, Rochester, NY 14642, USA

**Keywords:** Anti-bacterial surfaces, Nano- and microstructures, Fs-LIPSSs, Superhydrophobic surfaces

## Abstract

Femtosecond laser-induced surface structuring is a promising technique for the large-scale formation of nano- and microscale structures that can effectively modify materials’ optical, electrical, mechanical, and tribological properties. Here we perform a systematic study on femtosecond laser-induced surface structuring on gold (Au) surface and their effect on both hydrophobicity and bacterial-adhesion properties. We created various structures including subwavelength femtosecond laser-induced periodic surface structures (fs-LIPSSs), fs-LIPSSs covered with nano/microstructures, conic and 1D-rod-like structures (≤ 6 μm), and spherical nanostructures with a diameter ≥10 nm, by raster scanning the laser beam, at different laser fluences. We show that femtosecond laser processing turns originally hydrophilic Au to a superhydrophobic surface. We determine the optimal conditions for the creation of the different surface structures and explain the mechanism behind the formed structures and show that the laser fluence is the main controlling parameter. We demonstrate the ability of all the formed surface structures to reduce the adhesion of Escherichia coli (E. coli) bacteria and show that fs-LIPSSs enjoys superior antibacterial adhesion properties due to its large-scale surface coverage. Approximately 99.03% of the fs-LIPSSs surface is free from bacterial adhesion. The demonstrated physical inhibition of bacterial colonies and biofilm formation without antibiotics is a crucial step towards reducing antimicrobial-resistant infections.

## Highlights

• We present a detailed study on the formation of various surface structures formed using femtosecond laser treatment.
• We show that femtosecond laser processing turns originally hydrophilic Au to super-hydrophobic surface.
• We demonstrate the ability of the treated surface to reduce the adhesion of E. coli bacteria.

## 1 Introduction

**Fig. 1:**
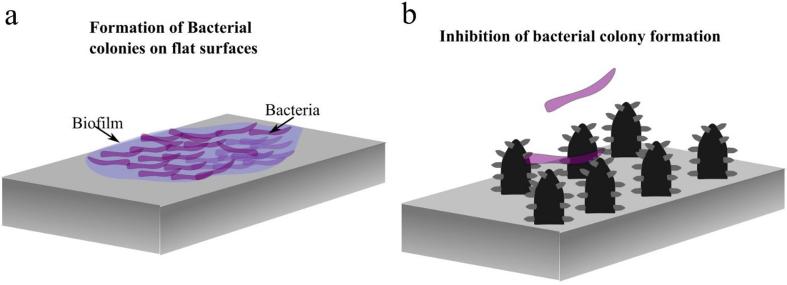
Schematics showing (a) the formation of bacterial colonies and a bacterial biofilm on a flat surface. (b) On the other hand, the creation of surface micro/nanostructures inhibits the formation of bacterial colonies.

Bacteria and other microorganisms form biofilms by first attaching to a surface, forming conglomerates, and eventually developing extracellular polymeric substances matrix [1], [2]. Once bacteria form biofilms their eradication using antibiotics becomes considerably more difficult. The effect of antibiotics is usually limited to the top layer of the biofilm, while bottom layers are protected and eventually develop antibiotic resistance [3] (see Fig. 1a). Antimicrobial-resistant infections are expected to claim up-to 10 million lives by 2050 a year [3]. Accordingly, the need to develop antibacterial surfaces that prevent the ab initio formation of bacteria is of paramount importance particularly in devices and equipment that can transfer pathogens, i.e., medical equipment, food containers and personal electronics, etc.

Naturally occurring anti-bacterial surfaces, e.g., Cicada wing, Gecko skin, and Dragonfly wings, effectively kill certain types of bacteria [3]. Biomimetic surface functionalization of antibacterial surfaces aims to create nano- and microscale structures via chemical etching, plasma-spray, grit blasting, pulsed laser irradiation, laser ablation in liquids and photochemical reduction of surface processing [4], [5], [6]. Structures with nanoscale dimensions (50 nm–250 nm) [3] can act as a bactericidal surface which pierces through bacterial cell walls, while larger structures (~0.5 µm−5 µm) are optimal for minimizing bacterial adhesion which decreases the possibility of forming bacterial colonies (Fig. 1b) [7], [8], [9], [10]. Furthermore, surfaces with nano/microscale hierarchical structures were shown to strongly reduce the bacterial adhesion on surfaces and inhibit the formation of bacterial biofilms and colonies [11]. This is because surface structures prevents bacteria from generating attractive radial fluid flow that leads to cluster formation [11], [12], [13]. Superhydrophobic surfaces also have antibacterial adhesion properties as they naturally have nano/microscale structures in addition to the repulsion of water which limits the access of bacteria on the superhydrophobic surface [10], [14]. Using chemical methods, fluorinated silica colloid-coated superhydrophobic surfaces with a contact angle (θ_M_) of 167°, reduced the adhesion of Staphylococcus aureus (S. aureus) and Pseudomonas aeruginosa (P. aeruginosa) [15]. Similarly, chemical vapor deposition technique was employed to create a superhydrophobic surface on silicone elastomers with θ_M_ of 165° that reduces the adhesion of E. coli and S. aureus to 63% and 58%, respectively [16].

In the context of biomedical applications, several nanosecond and femtosecond laser-induced surface structures have been studied for reducing bacterial adhesion, retention and colony formation of different types of bacteria. For instance, micro- and nanoscale quasi-periodic self-organized structures were produced on titanium to reduce the adhesion of P. aeruginosa, and S. aureus [17], fs-LIPSSs on titanium were shown to reduce the adhesion of S. aureus [18]. In addition, the formation of superhydrophobic micro papillae patterns covered with nanostructures on steel by picosecond laser pulses minimized the adhesion of E. coli and S. aureus [19]. Moreover, hierarchical structures were produced using picosecond laser on stainless steel to reduce the adhesion of E. coli [20], micro-spikes, fs-LIPSSs and nano-pillars using fs-laser pulses on steel reduced the retention of E. coli and S. aureus [21]. Similarly fs-LIPSSs on steel were created by femtosecond laser pulses that resists the biofilms formation of E. coli and S. aureus [22].

On the other hand, Au enjoys high biocompatibility due to its high resistance to corrosion in oxygen-rich environment, low toxicity and chemical inertness [23]. Due to these properties, Au is employed for a range of medical applications [24]. For instance, Au is used for dental implants and in medications for treating depression, epilepsy, migraine, amenorrhea and impotence among others [24], [25]. Moreover, Berry et al. [26], performed a comparative study on several metals (gold, titanium, chromium, cobalt, iron, and aluminum ) for in vitro antibacterial activity for dental implants applications and showed that gold has the strongest antibacterial performance. In addition, Au nanoparticles showed the strong antibacterial activity of E. coli [27]. However, direct surface structuring of Au is not demonstrated for bacterial adhesion.

In this work, we demonstrate a single-step method for fabricating a range of novel surface structures on Au by using fs-laser pulses. We created various structures including subwavelength fs-LIPSSs, fs-LIPSSs extensively covered with nano/microstructures, microscale structures including conic and 1D rod-like structures in the range of ≤6 μm and spherical nanostructures with a diameter ≥10 nm at different laser fluences. We determined the optimal conditions for the formation of each type of structures and showed that the laser fluence is the main controlling parameter. We show that fs-laser processing turns originally hydrophilic Au to superhydrophobic surface. We test the adhesion of E. coli bacteria on the fs-laser structured Au compared to unstructured Au. We demonstrate the ability of all the formed surface structures to reduce the adhesion of E. coli bacteria. In particular, Au surface with fs-LIPSSs shows the best antibacterial adhesion properties with only 0.97% of the fs-LIPSSs surface covered with bacterial colonies.

## 2 Experimental details

### 2.1 Sample fabrication

**Fig. 2:**
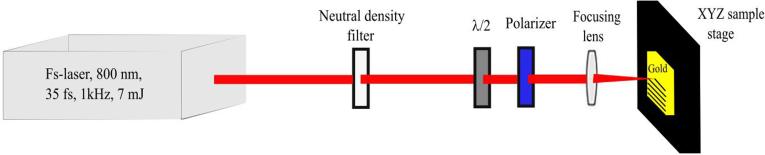
The experimental setup for fs-laser treatment of Au surface by raster scanning the Au sample target. A combination of polarizer and half-waveplate is used to control the power of incident beam while a focusing lens is used to focus the laser beam on the target.

To fabricate micro/nano structures on a gold surface, we used an Astrella integrated Ti: Sapphire amplifier femtosecond laser from coherent, as an irradiation source to deliver horizontally polarized pulse trains at the repetition rate of 1 kHz, with a central wavelength of λ = 800 nm and a pulse duration of τ = 30 fs, as shown in the Fig. 2. The maximum pulse energy delivered by the laser system is 7 mJ, which was attenuated using a combination of half-waveplate and a linear polarizer. The sample was mounted at a computerized XYZ precision stage. The laser was incident at normal incidence using a lens of focal length 20 cm and the focal spot diameter was 120 µm. First, under fixed-spot irradiation, we study the damage threshold on Au surface, which is experimentally obtained to be 0.08 J/cm^2^. The number of shots were controlled by using an electromechanical shutter. To determine the effect of surface structuring on wetting and antibacterial properties, we heavily optimized the laser processing parameters, such as scanning speed from V = 0.01–2 mm/s, focal spot distance of the laser beam from 0 to 600 μm and interspacing between two scanned lines from 50 μm to 150 μm. The optimal results are obtained by raster scanning the laser beam at 0.7 mm/s across the sample area of 3 × 3 mm^2^, interspacing between two adjacent lines are kept as 100 µm, and the target was placed at 600 μm before the laser focal plane. By fixing these laser parameters, we varied the laser fluence from 0.1 J/cm^2^ to 3 J/cm^2^ and fabricated 15-samples for investigating the response of bacterial adhesion and water contact angle. A bulk circular disks of Au (obtained from Goodfellow, Ltd. company with 99.99% purity) was used as a target material due to its extensive biomedical and plasmonic applications [28]. The laser fluence (*F*) was varied by increasing the power. After the laser micro/nano structuring process, the surface morphology was analyzed by Scanning Electron Microscopy (SEM/FIB, Zeiss – Auriga) and three-dimensional (3D) laser scanning microscope.

### 2.2 Contact angle measurements

Following fs-laser-induced micro/nanostructuring, we performed contact angle measurements with an optical contact angle measuring system (a VCA 2500XE video contact angle system) to study the wetting properties of untreated and laser-treated surfaces. For measuring θ_M_, we used the sessile-drop method that determines the advancing θ_M_ in a few seconds from the moment when a water drop is brought into contact with the surface. A distilled water droplet with approximately 1 µl volume was carefully placed on the untreated and treated surfaces using a computer-controlled stage. The θ_M_ is measured from the sideview of the captured photographs. All experiments were carried out in an ambient environment of 1 atm. Each experimental data point is the average of three measurements and the θ_M_-error is the standard deviation.

### 2.3 Bacterial adhesion test

After laser-texturing, the samples were ultrasonically cleaned for 20 min. Before the bacterial adhesion test, all the samples were sterilized by high temperature sterilization (220 °C, 2 h) to avoid external bacterium affecting the experimental results. DH5α strain was taken from glycerol stock, stored at −80 °C, and grown overnight in 10 ml Luria- Bertani (LB) medium with 10 µl ampicillin (100 mg/ml) at 37 °C under gentle shaking of 80–100 rpm. Overnight culture was then adjusted to an optical density of 0.02 (OD_600_) with approximate cell number of 1.6 × 10^7^/ml. Now this overnight cultured media, having approximately 0.02 OD, was further incubated with our laser fabricated samples and control in 250 ml conical flask at 37 °C under gentle shaking of 80–100 rpm for 24 h and samples were proceeded for SEM. In our experiments, we have 5 different surface structures in triplicates, i.e., the bacterial adhesion tests were done on 15 samples.

### 2.4 SEM analysis

After 24 h incubation, samples were washed with phosphate-buffered saline (PBS) to remove the loosely adherent bacteria and fixed in 10% paraformaldehyde for 30 min at 4-degree and dried with nitrogen before performing SEM to examine the morphologies of bacterial colonies.

### 2.5 Bacterial quantification

The area covered by bacterial colonies is quantified using ImageJ (v1.52a Wayne Rasband, NIH USA) software [29] which is conventionally used to measure particle dimensions similar to previously shown in [7]. Three images per treated and untreated samples were evaluated. We first select regions from the SEM surface image where bacteria are present. We then count the bacterial adhesion for an area of 20 × 20 µm^2^ for all the fluences and control (unstructured sample). Afterwards, we normalized the total area coverage by comparing it with untreated sample. The surface area covered with bacterial colonies is normalized to the median colonized surface of the control area (unstructured area).

## 3 Results and discussion

**Fig. 3:**
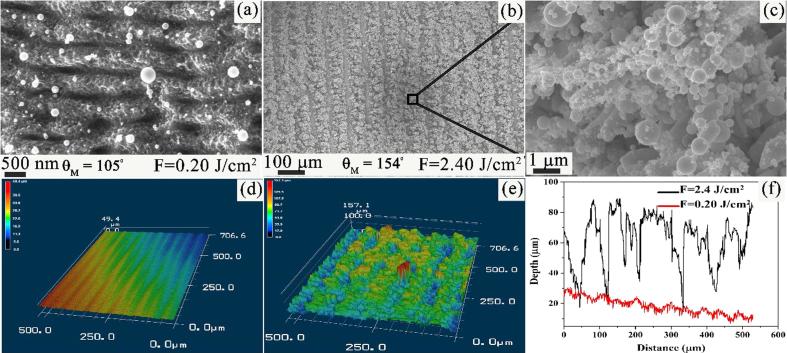
SEM images of fs-LIPSSs and nano/microstructures at the fluence of (a) *F* = 0.20 J/cm^2^ and (b-c) *F* = 2.4 J/cm^2^ at normal incidence, respectively. 3D optical images of the Au surface with (d) fs-LIPSSs and (e) spherical nanostructures, respectively. (f) Cross-sectional line profile of the modulation depth for the structure.

Irradiating the Au surface with fs-laser pulses at various fluences creates highly ordered structures (fs-LIPSSs) which evolves as a function of laser fluence to highly disordered surfaces with random nano/microstructures. The surface morphologies and corresponding depth profiles of the highly ordered and highly disordered surfaces are shown in Fig. 3. Fig. 3(a) demonstrates the formation of fs-LIPSSs, oriented perpendicular to incident laser polarization with a subwavelength periodicity of Λ = (577 ± 37) nm at the fluence of *F* = 0.20 J/cm^2^. Fig. 3(b) shows SEM image of the scanned lines at the fluence of *F* = 2.4 J/cm^2^, where distinct ablated microgrooves and surface structures are formed. The clusters of dense spherical nanostructures are shown in Fig. 3(c). The corresponding 3D optical images of fs-LIPSSs and spherical nanostructures are depicted in Fig. 3(d) and Fig. 3(e), respectively. The average modulation, peak-to-valley, depth of fs-LIPSSs and nano/microstructures is shown in the cross-sectional profile of Fig. 3(f). The θ_M_ for fs-LIPSSs and nano/ microstructures was measured to be 105° and 154°, respectively. We note here that a surface is referred to as hydrophobic when θ_M_ > 90° and superhydrophobic when water forms θ_M_ ≥ 150°, with only a sliding angle of few degrees (<10°) [30].

**Fig. 4:**
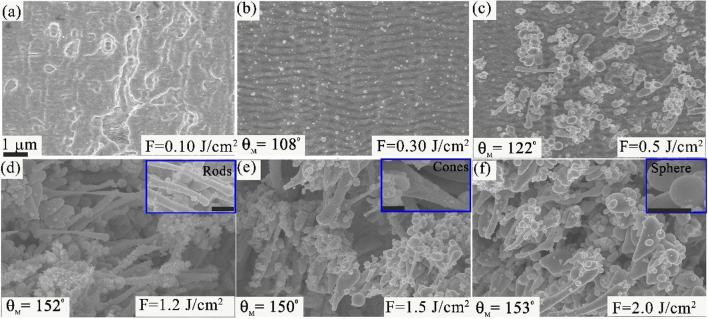
SEM images showing the evolution of laser-induced surface structures with varying the laser fluence. (a) SEM images of random roughness at *F* = 0.1 J/cm^2^, (b) fs-LIPSSs at *F* = 0.30 J/cm^2^, (c) fs-LIPSSs covered with nano/microstructures at *F* = 0.5 J/cm^2^, a combination of 1D rods, conical and spherical structures at (d) *F* = 1.2 J/cm^2^, *F* = 1.5 J/cm^2^, and *F* = 2.0 J/cm^2^. The measured values of θ_M_ for different surface structures are shown. The insets of Fig. 4(d-f) are the rods, cones and spherical structures, respectively. The scale-bar in the insets is 500 nm. The scale-bar of 1 µm shown in Fig. 4(a) is same for Fig. 4(a-f).

To study the effect of fluence in the evolution of surface morphology, we varied the laser fluence from *F* = 0.1–3.0 J/cm^2^. Initially, random roughness/scratches are formed at *F* < 0.1 J/cm^2^ [Fig. 4(a)]. The formation of uniform fs-LIPSSs structures are observed at *F* = 0.30 J/cm^2^ [Fig. 4(b)]. At intermediate fluences, 0.5 < *F* < 0.8 J/cm^2^, fs-LIPSSs extensively covered with nano/microscale structures are produced [Fig. 4(c)]. Therefore, by changing the laser fluence, we can control the surface morphology. Fs-LIPSSs do not form for laser fluences above 1 J/cm^2^. For the fluence range 1 < *F* < 2 J/cm^2^, 1D rods, cones, spherical nanostructures and redeposited nanoparticles are the dominant structural features [Fig. 4(d–f)].

**Fig. 5:**
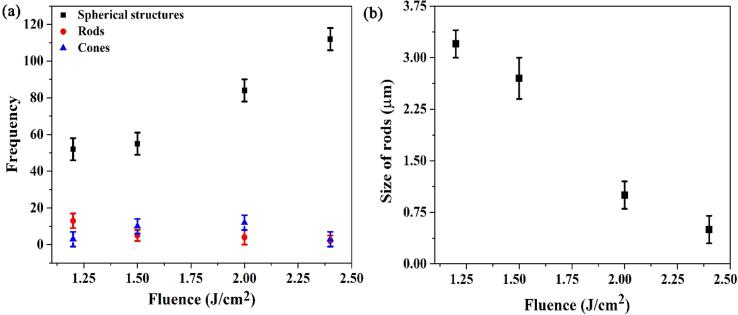
(a) The measured frequency of laser-induced spherical, 1D rods and conic structures shown earlier in Figs. 4(d-f) and Fig. 3(c). (b) The average size of 1D rod structures as a function of laser fluence. The increase in spherical structure frequency as a function of laser fluence corresponds to a decrease in the rod size. This observation indicates that higher fluence lead to fragmentation of the formed rods.

Fig. 5(a) demonstrates the change in the frequency of 1D rods, cones and spherical nano/microscale structures as a function of laser fluence. The size of 1D rods decreases with increasing the laser fluence as shown in Fig. 5(b). Similarly, rods size also decreases from 3.2 µm [Fig. 4(d)] to 1 µm [Fig. 4(f)] with the increase in laser fluence. On the other hand, more spherical structures are formed at higher fluences [Fig. 5(a)], which indicates that the reduction in the rod’s frequency and size is due to laser fragmentation. For Fig. 5(a) and Fig. 5(b), the error bars are calculated as the standard deviation of the size of multiple 1D rods, cones and spherical structures by inspecting the SEM images using imageJ software. The frequency of spherical, 1D rods and conic structures at different laser fluences are obtained from 5 × 5 µm^2^ in Figs. 4(d-f) and Fig. 3(c).

**Fig. 6:**
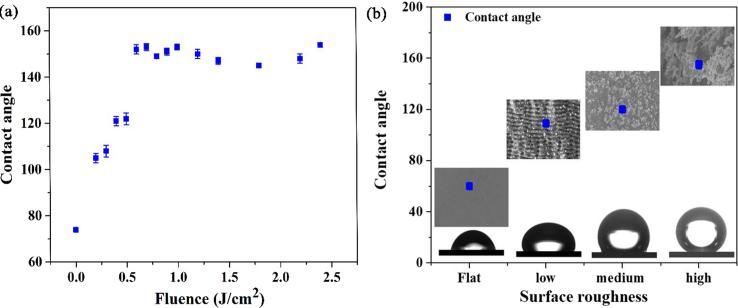
(a) The measured contact angle values as a function of laser fluence. (b) Contact angle values measured on the initial surface roughness at low fluence, low, medium and highly-dense surface structures and corresponding surface morphologies are depicted in the insets.

The variation in θ_M_ measured for 15 samples irradiated at different laser fluences are presented in Fig. 6(a). The increase in θ_M_ for water droplets placed on different surface structures along with different surface morphologies are shown in Fig. 6(b). The water contact angle measurement shows that fs-LIPSSs treatment of Au turns its originally hydrophilic untreated surface [θ_M_ ~ 74°] to hydrophobic surface, i.e., fs-LIPSSs [θ_M_ ~ 108°]. While the nanostructures covered fs-LIPSSs further enhance the hydrophobicity to θ_M_ ~ 122. The θ_M_ measurements reveal a remarkable variation among the different surface morphologies, indicating that as the surface nano/microstructures increases, the θ_M_ significantly increases as well. Clearly, at F ≥ 1.2 J/cm^2^, the Au surface covered with nano/micro rods, cones and spherical structures are superhydrophobic (θ_M_ ≥ 150°). We note here that spherical, conic and rods-like structures show stronger hydrophobic behavior as compared to fs-LIPSSs [see the values of θ_M_, in Fig. 4(d–f)].

**Fig. 7:**
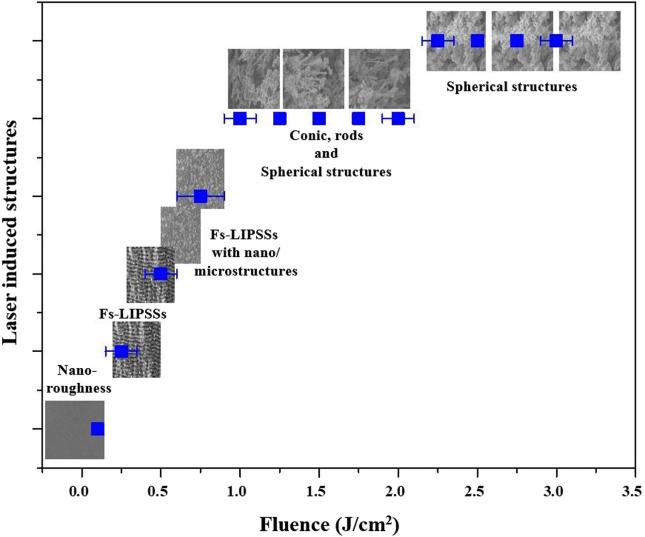
Laser-induced structural features as a function of laser fluence. The fluence range for each structural feature is estimated and error bars are calculated as the standard deviation in variation of fluence range.

Fig. 7 summarizes the optimal conditions for the creation of different kinds of surface structures, as a function of laser fluence. Uniform fs-LIPSSs are formed at a fluence of *F* = 0.30 J/cm^2^. As we increase the laser fluence, surface structures change from fs-LIPSSs to fs-LIPSSs covered with nano/microstructures for *F* = 0.50 J/cm^2^. The surface structures evolve to a combination of 1D rods, conic and spherical structures for *F* = 1.2 J/cm^2^, and finally spherical structures are formed at *F* = 2.0 J/cm^2^.

### 3.1 Physical mechanism behind the formation of different Au surface structures

Generally, LIPSS formed with nanosecond laser have a period equivalent to incident laser wavelength. However, fs-LIPSSs differs from nanosecond LIPSS in two aspects. First, the period of fs-LIPSSs is slightly shorter than the incident wavelength [31], and secondly, fs-LIPSSs are covered extensively with nano/microscale structures. The formation of fs-LIPSSs on solids is generally understood in the framework of scattered surface wave interference theory [32], [33], [34], [35]. Briefly, the irradiation of a metallic surface with fs-laser pulses excites surface waves, known as surface plasmon polaritons (SPPs), that interfere with the incident pulse resulting in a spatial distribution of the field intensity across the material surface which leads to selective, periodic ablation. For a metal, the periodΛ of fs-LIPSSs is given by [36];

(1) Λ=λ1/Reη±sinθ

where λ*_I_* is the incident wavelength, η is the effective refractive index of the dielectric-metal interface for surface plasmons and is given by; η=εmReεD/εmRe+εD1/2,εmRe is the real part of metallic dielectric constant and *ε*_D_ is the dielectric constant of the ambient environment. The formation of random nano/microstructures is due to the Marangoni-driven flow, that arises due to surface tension gradient in the molten layers, caused by high-intensity femtosecond laser pulses [37], [38], [39], [40].

### 3.2 The impact of structural features/dimension on bacterial adhesion

**Fig. 8:**
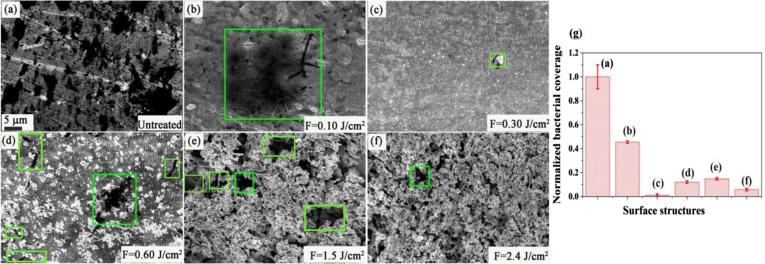
Representative E. coli attachment and biofilms formation on the unstructured (control) and laser-induced structured samples at different fluences after 24 h incubation. (a) SEM image of bacterial colonization and biofilms (black areas) on the untreated Au surface. (b-f) Bacterial colonization on the structured surfaces treated at different fluences. The scale-bar of 5 µm is same for (a-f). Clearly, the structured surfaces prevent the formation of bacterial colonies. The green frames shown are the region for bacterial colonization. (g) The normalized surface coverage for E. coli cells after 24 h on unstructured and laser-structured samples. The values are normalized to the colonized surface on the unstructured surface. (For interpretation of the references to colour in this figure legend, the reader is referred to the web version of this article.)

We now study the efficacy of the formed surface structures in inhibiting bacterial adhesion of E. coli. As we discussed earlier, the mere existence of surface structures with dimensions smaller than that of a given bacteria limits its ability to form a colony. We investigate the growth of bacteria on all the Au surfaces obtained via fs-laser processing and compare the results to unprocessed Au. Fig. 8 shows SEM images of unstructured [Fig. 8(a)] and structured [Fig. 8(b-f)] Au at different fluences. Dark regions delineated with green frames in Fig. 8 are bacterial colonies.

**Fig. 9:**
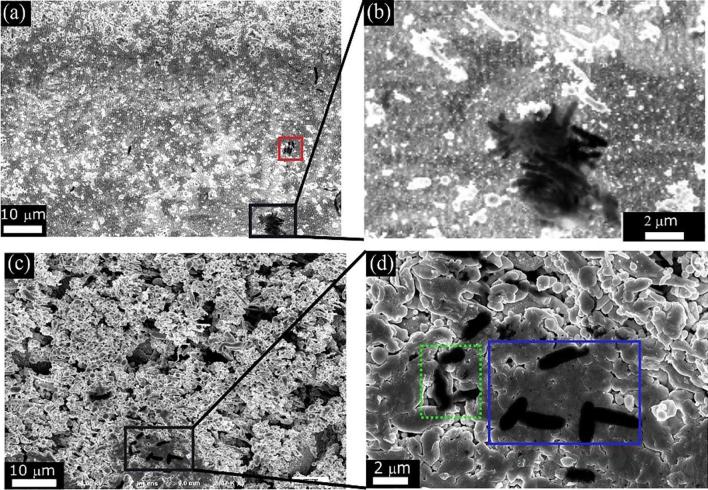
The adhesion of individual bacteria and large bacterial colonies formation on fs-LIPSSs covered with nano/microstructures in (a). (b) The magnified view of bacterial colonies on nanostructure covered fs-LIPSSs. (c) Bacterial colonies are observed for a combination of conic, rods and spherical structures, (d) and a magnified view of the surface clearly showing E. coli bacteria residing in the flat regions of the structured surfaces. In both cases, the colonies are formed within the non-structured regions.

Except for surfaces with only random nano-roughness (formed using F < 0.1 J/cm^2^), all structures significantly reduced the formation of bacterial colonies [Fig. 8(b)]. Fs-LIPSSs shows the strongest antibacterial adhesion properties [Fig. 8(c)] with 99.03% reduction in bacterial colonies compared to untreated Au surface (dark regions). Fs-LIPSSs extensively covered with nano/microstructures shows less efficient antibacterial adhesion properties with 90% reduction in bacterial colonies [Fig. 8(d)]. A low magnified and corresponding enlarged view of bacterial colonies can be seen in the Fig. 9(a-b). For Au surface with a combination of conic, rods and spherical structures, we observed the formation of bacterial colonies in regions that are not covered with surface structures [Fig. 8(e)] and also see Fig. 9(c–d) where bacteria are attached to non-structured regions. Consequently, the antibacterial performance of such surfaces is lower than the case of fs-LIPSSs and is ~87.5%. Note that for completely flat surfaces delineated with blue rectangles in [Fig. 9(d)], we see E. coli bacteria with their expected dimensions. However, for other regions (delineated with a green dotted rectangles) the bacteria are distorted. This could be due to physical rupture of the bacteria due to the existence of nanostructures [3].

The surface with dense spherical structures (for *F* > 2 J/cm^2^) shows improved antibacterial adhesion properties of 95.2% as shown in Fig. 8(f). Fig. 8(g) shows the normalized surface coverage for E. coli cells on unstructured and laser-structured samples.

We note here that fs-LIPSSs enjoys less hydrophobicity compared to other surface structures that showed lower antibacterial adhesion properties, e.g., fs-LIPSSs with extensive nano/microstructures. Although it is expected that superhydrophobic surfaces show better antibacterial adhesion [17], fs-LIPSS shows better antibacterial adhesion compared to other surfaces with higher hydrophobicity. This is because bacterial colony inhibition depends also on the ability of the bacteria to create radial fluid flows that lead to cluster formation [19]. Fs-LIPSSs provide large surface coverage of structures that limit bacteria clustering. This is because fs-LIPSSs are periodic surface structures with a specific period (in this work ~ 577 nm) that covers the entire treated region. On the other hand, randomly distributed nano/microstructures have pockets where bacteria can form colonies. This is further illustrated in Fig. 9(c–d), where bacteria are formed in the gaps between structures.

## 4 Conclusion

We performed a detailed study on the surface structuring of Au surface by the irradiation of ultrafast femtosecond laser pulses. We found that various structures, such as subwavelength fs-LIPSSs, fs-LIPSSs covered with nano/microstructures, conic and 1D-rod-like structures (in the range of ≤6 μm), and spherical nanostructures with a diameter ≥10 nm can be produced by optimizing the laser processing parameters. We showed that femtosecond laser-induced surface structures turned hydrophilic Au to superhydrophobic surface. We demonstrate the ability of all the formed surface structures to reduce the adhesion of E. coli bacteria, in contrast to untreated/control surface, and show that fs-LIPSSs enjoys superior antibacterial performance. This is because fs-LIPSSs are periodic surface structures with a specific period (in this work ~ 577 nm) that covers the entire treated region. On the other hand, other random surface structures formed via fs-laser ablation allow for relatively flat regions with no surface structures which allows for the formation of bacterial colonies. Based on experimental results, we have determined the optimal conditions for the formation of each type of structure. The texturing of Au surface with the observed dimensions is particularly important for Au to enhance bacteria repellency.

## Author Contributions

S. A. J, M. A, M.E. and C.G. discussed and designed the project. S.A.J performed the laser fabrication. S.A.J., and M. E, performed the SEM and contact angle measurements. S. A. J and S. C. S performed laser scanning microscopy. J. A. B. and J. J. H performed the antibacterial tests. S.A.J., M.A, M.E, and C.G. performed data analyses. S.A.J., M.A, M.E., and C.G. wrote the paper. All the authors commented on the paper.

## Declaration of Competing Interest

We declare that we have no conflict of interest.
